# Evaluation of Glycemic and Lipid Profile of Offspring of Diabetic Wistar Rats Treated with *Malpighia emarginata* Juice

**DOI:** 10.1155/2011/173647

**Published:** 2011-01-23

**Authors:** Sandra M. Barbalho, Débora C. Damasceno, Ana Paula Machado Spada, Miréia Palhares, Karla Aparecida Martuchi, Marie Oshiiwa, Viviane Sazaki, Vanessa Sellis da Silva

**Affiliations:** ^1^Medical School of Medicine of Marília (UNIMAR), Faculty of Technology and Foods of Marília, Avenida Higino Muzzi Filho, 1001, Marília, SP 17525902, Brazil; ^2^Laboratory of Experimental Research on Gynecology and Obstetrics, Botucatu Medical School, UNESP, Distrito Rubião Junior, S/N, Botucatu, SP 18618000, Brazil; ^3^Biochemistry Department, Faculty at UNINOVE and SENAC, USP/São Paulo, SP 01041000, Brazil; ^4^Nutrition Department, Methodist University of Piracicaba, UNIMEP, Rua Tenente Florencio Pupo Neto, 300, Lins, SP 16400680, Brazil; ^5^Nutrition Department, Frigorificos Bertin S/A, Rodovia de Acesso Lins-Getulina s/n, Parque Industrial, Lins, SP 16400000, Brazil; ^6^Faculty of Technology and Foods of Marília, FATEC, Avenida Castro Alves, 62, Marília, SP 17506000, Brazil

## Abstract

Knowing that maternal diabetes is related to hyperglycemia and fetal hyperinsulinemia, which affect the lipid metabolism, the aim of this study was to evaluate the effects of *Malpighia emarginata* (acerola) juice on the glycemic and lipid profile of offspring of diabetic and nondiabetic Wistar rats. The adult offspring of non-diabetic dams and of dams with severe streptozotocin-induced diabetes were divided into groups: G1, offspring (of control dams) treated with water, G2, offspring (of diabetic dams) treated with water, G3, male offspring (of control dams) treated with acerola juice, and G4, male offspring (of diabetic dams) treated with acerola juice. The offspring of diabetic dams treated with acerola juice showed significantly decreased levels of glucose, cholesterol, triglycerides, and increased HDL-c. The use of acerola juice is a potential strategy to aid in the prevention of DM and dyslipidemia and its complications or to act as an auxiliary in the treatment of these diseases.

## 1. Introduction

Diabetes mellitus is a disease characterized by chronic hyperglycemia because of a total or relative lack of insulin [1].

Among the many groups of people who suffer from this disease are gestating women. Gestational diabetes leads to modifications in the metabolism of the mother and her offspring caused by the mother's hyperinsulinemia and hyperglycemia. These conditions affect the fetal metabolism during the gestational period and extend throughout the life of the offspring [2, 3].

Maternal hyperinsulinemia and hyperglycemia give rise to complications such as macrosomia, spontaneous termination of pregnancy, premature birth, and metabolic and respiratory complications in the newborn [4, 5].

The presence of obesity and diabetes is more frequent in childhood and adolescence when there is a history of maternal or gestational diabetes. In these conditions, metabolic disorders can affect the growth and metabolism of descendants and subsequent generations [6, 7].

There are many allopathic medications for controlling glycemia and dyslipidemia but they are usually expensive and have been implicated in congenital anomalies [8]. Due to the high cost of medications, pregnant women turn to alternative remedies to treat diabetes and other diseases, motivating the search for alternatives that are normally easier to find and inexpensive. In view of this tendency, there is a need for studies to determine the effects of medicinal plants and phytotherapeutic products and to establish their ideal therapeutic schemes to take advantage of their benefits and reduce the occurrence of adverse effects. In Brazil and many parts of the world, medicinal plants are sold in open street markets and grown in the backyards of private homes [9, 10].

The acerola tree (*Malpighia emarginata*) is a very popular plant in South America, and its fruit is known to be rich in ascorbic acid and polyphenols. Hanamura et al. [11] studied the antihyperglycemic effects of polyphenols from acerola fruit and found positive effects. However, there are no reports in the literature about the effects of *Malpighia emarginata* on the glycemic and lipid profile in the offspring of streptozotocin-induced diabetic Wistar rats. 

Knowing that maternal diabetes is related to hyperglycemia and fetal hyperinsulinemia, which affect the lipid metabolism, the aim of this study was to evaluate the effects of *Malpighia emarginata *(acerola) juice on the glycemic and lipid profile of the offspring of diabetic and nondiabetic Wistar rats. 

## 2. Material and Methods

### 2.1. Parental Generation

The animals included in this study were treated according to the “Guide to the care and use of experimental animals”, which delineates the principles by the Canadian Council for the care to laboratory animals. The study was initiated after its approval by the Ethics Committee under registration number 2500000764/2007-47. Wistar rats were kept in the vivarium of our university under controlled conditions (12/12-hour light/dark cycle, ambient temperature of 22 ± 2°C, relative humidity of 60 ± 5%, and water and chow ad libitum). 

### 2.2. Diabetes Induction

Nondiabetic female rats (*n* = 20) weighing approximately 250 g underwent a seven-day adaptation period in the room where the experiment was designed. After such period, randomly selected rats received the intravenous administration (caudal vein) of 40 mg of streptozotocin/Kg (STZ—SIGMA Chemical Company, St. Louis, MO, USA) diluted in citrate buffer (0.1 M; pH 4.5). Nondiabetic animals received a similar volume of citrate buffer by the same route of administration of the diabetogenic drug [12]. For criteria of inclusion, the rats given citrate buffer and presenting with glycemia <120 mg/dL at day 0 of pregnancy were included in the nondiabetic groups. Rats from the STZ group presenting with glycemia 300 mg/dL at day 0 of pregnancy were included in the diabetic groups. 

### 2.3. Mating Period

All female rats were mated overnight to nondiabetic male rats untreated with acerola juice. The mornings that spermatozoa were found in the vaginal smear were designated gestational day 0. The mating procedure continued for 15 consecutive days, which comprises approximately three oestral cycles, and until replicate numbers were obtained for each group (*n* = 15 animals/group). Nonmated female rats in this period were considered infertile and discounted from the study [13]. 

### 2.4. Pregnancy Period

Glycaemic level (food ad libitum overnight) was monitored during pregnancy in the morning using the glucose oxidase method [13] at days 0, 7, 14, and 21 of pregnancy in all experimental groups. Approximately at the day 21 of pregnancy, after birth of the offspring, the dams remained in individual cages with their offspring until weaning (21 days). Food intake was addressed every day and body weight once a week. 

### 2.5. Offspring

After weaning, the offspring (60 males) were kept in collective cages until they reached adulthood. The adult animals, weighing approximately 250 g, were divided into 4 experimental groups: G1 (*n* = 15), male offspring (from control dams) treated with vehicle (water), G2 (*n* = 15), male offspring (from diabetic dams) treated with vehicle (water), G3 (*n* = 15), male offspring (from control dams) treated with *Malpighia emarginata *(acerola) juice, and G4 (*n* = 15), male offspring (from diabetic dams) treated with *M. emarginata *(acerola) juice.

The animals from groups G3 and G4 received *Malpighia emarginata *(acerola) juice at a dose of 0.58 g/kg once a day (at early morning) for 30 consecutive days. The dose administered to the animals was based on 200 g/L, which corresponds to the daily intake of 200 mL of juice by an adult man weighing 70.0 Kg (this consumption was based on popular consultation to prepare the juice). 

Food intake was addressed every day and body weight once a week. 

### 2.6. Acerola Juice Preparation

Acerola fruits (obtained from Cooperativa Agrícola de Lins-SP (COALINS)) were washed, weighed (200 g/L), and triturated with water in a blender for 2 minutes. The juice was filtered and administered daily. It was prepared every day 15 minutes before its administration. 

### 2.7. Blood Collection and Biochemical Profile Determination

At the end of the treatment (30 days), the offspring from diabetic and non diabetic dams (60 male rats) were anesthetized with sodium pentobarbital (150 mg/kg) and killed. Blood samples were collected in order to determine the biochemical profile (total cholesterol, HDL-c, LDL-c, triglycerides, and glucose). Tests were performed according to the methodology proposed by commercial kits: LABTEST (Lagoa Santa, Belo Horizonte, MG) for glycemia, total cholesterol and HDL-c, and triglycerides and WIENER LAB (São Paulo, SP) for LDL-c. The results were interpreted according to the criteria established by the American Diabetes Association [1]. 

### 2.8. Statistical Analysis

Data analysis was performed by using Students *t*-test, and the level of significance adopted was 5%. 

## 3. Results

The following 4 tables show the results of the biochemical profile of the offspring from nondiabetic and diabetic dams.

Table 1 compares G1 (offspring of nondiabetic dams treated with water) and G2 (offspring of diabetic dams treated with water). Male offspring of diabetic dams (G2) presented significantly higher levels of glycemia, triglycerides and total cholesterol and significantly lower HDL-c levels than G1.

Table 2 compares G1 (offspring of control dams), that received water, and G3 (offspring of control dams), treated with acerola juice. As can be seen, the male offspring treated with acerola juice showed significantly higher HDL-c levels.

Table 3 shows that there are no differences in the biochemical profile of G3 (nondiabetic offspring treated with acerola juice) and G4 (diabetic offspring treated with acerola juice).

A comparison of G2 (offspring of diabetic dams), treated with water, and G4 (offspring of diabetic dams), treated with acerola juice, suggests that the use of acerola juice reduced glycemia, triglycerides, total cholesterol, and LDL-c and increased HDL-c levels (Table 4).

No differences were found in food intake and body weight among the groups at the beginning and end of the treatment (Table 5). 

Table 6 shows that the food intake of nondiabetic and diabetic dams on the first day of pregnancy and on the day preceding the birth of their offspring did not differ significantly among the groups. The mothers showed a significant weight increase at the beginning and end of gestation, but no significant differences in weight were found between diabetic and nondiabetic mothers. 

## 4. Discussion

The results of this study reveal metabolic modifications in the offspring of diabetic dams when compared to offspring of nondiabetic dams. The animals (offspring of both diabetic and nondiabetic dams) treated with acerola juice presented higher levels of HDL-c and lower values of LDL-c. Modifications in levels of blood glucose and plasma fats are risk factors for metabolic syndrome and its future complications as vascular diseases [14–18]. The alterations resulting from maternal diabetes are related to hyperglycemia and fetal hyperinsulinemia, which affect lipid and protein synthesis [2, 4]. Furthermore, maternal hyperglycemia stimulates fetal growth due to the greater availability of glucose in the blood flow and the regulation of growth factors [4, 19]. In this work, the animals in the diabetic and nondiabetic groups did not show significant differences in body weight.

The results of this work indicate that the use of acerola juice can be beneficial in preventing and helpful in treating hyperglycemia and dyslipidemias in the offspring of diabetic dams. Numerous studies in the literature demonstrate that plants can also be used for this purpose in the offspring of diabetic dams [20–22]. Many other authors have reported the beneficial effects of plants on glycemic and lipid profiles. Baviloni et al. [23] showed the antihyperglycemic activity of stem bark extract of *Vatairea macrocarpa* in the treatment of diabetic rats. Badole et al. [24] found significant antihyperglycemic effects in alloxan-induced diabetic mice treated with *Pleurotus pulmonarius *and observed that it has potent synergistic antihyperglycemic effects when used in association with glyburide. Umar et al. [25] found that *Tetracera scandens* has antidiabetic efficacy in diabetic rats. Another study [26] observed a reduction in blood glucose and in the lipid profile of streptozotocin-induced diabetic rats treated with *Cassia glauca*. Bera et al. [22] observed that the effects of a polyherbal formulation (of eight medicinal plants) for the management of streptozotocin-induced diabetes in rats were comparable to those of the antidiabetic drug (Glibenclamide). Decreased lipids and glycemia were reported by Daisy et al. [27] using *Hunteria umbellata* and by Adeneye and Adeyemi [28] using isolated components of *Gymnema sylvestre*. Many other studies have demonstrated the beneficial effects of plants such as *Cynodon dactylon*, *Coriandrum sativum*, *Mentha piperita*, and *Amorphophallus konjac* on the glycemic and lipid profiles of diabetic rats (streptozotocin or alloxan-induced) [29–32]. Lin et al. [33] also showed that *Siraitia grosvenorii* polysaccharides can be helpful in reducing glucose and lipid levels in rabbits.

Acerola fruit is rich in vitamin C, calcium, iron, and polyphenols such as anthocyanins (cyanidin-3-alpha-O-rhamnoside and pelargonidin-3-alpha-O-rhamnoside), quercetin (quercetin-3-alpha-O-rhamnoside), chlorogenic and caffeic acids, and kaempferol [11, 34–36]. Kawaguchi et al. [37] described the flavonoid leucocyanidin-3-O-beta-D-glucoside and gave it the trivial name “aceronidin”, which possesses stronger antioxidative activity than alpha-tocopherol. Acerola also contains carotenoids that associated with bioflavonoids provide important nutrients and have a potential use as antioxidant. Brazil's climate and soil are very favorable for cultivating acerola, making this country the world's main producer of this fruit. Acerola is commercialized in different forms such as juices, jams, ice-creams, sweets, and liqueurs [38]. The presence of vitamins and antioxidants is known to reduce blood glucose, cholesterol, triglycerides, and LDL-c levels, and to elevate HDL-c levels [39–41]. Bhandari et al. [20] studied the antioxidant effects of *Embelia ribes* on streptozotocin-induced diabetes in Wistar rats and found that it has important antioxidant activity in combating free radicals produced in hyperglycemic conditions, thus protecting against loss of beta cells.

Flavonoids may act in distinct ways on various plasma lipid components [42], but the literature still does not explain the exact mechanisms whereby phenolic acids, flavonoids, and terpenoids exert these beneficial effects. The presence of diabetes may hasten the development of endothelial lesions and atherosclerosis, which may be minimized by the presence of antioxidants [43, 44].

Polyphenolic substances extracted from acerola can have inhibitory effects on maltase and *α*-glucosidase and reduce glucose absorption, which ends up in reducing glycemia [11]. Polyphenols also participate in preventing the formation of AGEs (advanced glycation products) in diabetics, which are implicated in many micro and macrovascular complications of diabetes mellitus [45]. The antioxidant properties of polyphenols prevent oxidative stress caused by the scavenging activity of free radicals and also show anti-inflammatory activities [46–48]. The results of this work indicate that acerola juice can be helpful in preventing future damage caused by diabetes and its complications, such as vascular diseases, which, worldwide, are among the main causes of death. 

## 5. Conclusion

The use of acerola juice is a potential strategy to aid in the prevention of DM and dyslipidemia and its complications or to act as an auxiliary in the treatment of these diseases.

Although the results of the use of acerola juice are promising, further studies are essential to evaluate its effects on human beings and to determine the ideal dosages to be used.

## Figures and Tables

**Table 1:** Biochemical profile of offspring treated with water (from nondiabetic dams: G1 and from diabetic dams: G2).

	Groups		*P* value
	G1	G2	
Glucose	67.7 ± 11.9	88.9 ± 11.6	.0002*
Triglycerides	119.9 ± 72.4	139.0 ± 92.5	.0000*
Cholesterol	76.8 ± 67.3	112.7 ± 24.7	.0131*
HDL-c	34.4 ± 3.3	30.4 ± 4.1	.0175*
LDL-c	65.0 ± 19.5	84.5 ± 37.1	.2676

Values presented as mean ± standard deviation (SD)/*significant values.

**Table 2:** Biochemical profile of offspring (from nondiabetic dams) treated with vehicle (G1) and offspring (from nondiabetic dams) treated with acerola juice (G3).

	Groups		*P* value
	G1	G3	
Glucose	67.7 ± 11.9	68.68 ± 23.75	.0771
Triglycerides	119.9 ± 72.4	83.00 ± 48.41	.2354
Cholesterol	76.8 ± 67.3	98.00 ± 22.70	.0610
HDL-c	34.4 ± 3.3	45.25 ± 19.98	.0000*
LDL-c	65.0 ± 19.5	57.36 ± 35.19	.0620

Values presented as mean ± standard deviation (SD)/*significant values.

**Table 3:** Biochemical profile of offspring treated with acerola juice (from nondiabetic dams: G3 and from diabetic dams: G4).

	Groups		*P* value
	G3	G4	
Glucose	68.68 ± 23.75	72.69 ± 16.09	.0637
Triglycerides	83.00 ± 48.41	87.47 ± 53.60	.3891
Cholesterol	98.00 ± 22.70	94.85 ± 16.73	.0604
HDL-c	45.25 ± 19.98	60.68 ± 13.43	.1047
LDL-c	57.36 ± 35.19	51.85 ± 13.6	.0556

Values presented as mean ± standard deviation (SD).

**Table 4:** Biochemical profile of offspring treated with vehicle (G2) and offspring treated with acerola juice (G4).

	Groups		*P* value
	G2	G4	
Glucose	88.9 ± 11.6	72.69 ± 16.09	.0000*
Triglycerides	139.0 ± 92.5	87.47 ± 53.60	.0000*
Cholesterol	112.7 ± 24.7	94.85 ± 16.73	.0335*
HDL-c	30.4 ± 4.1	60.68 ± 13.43	.0000*
LDL-c	84.5 ± 37.1	51.85 ± 13.6	.0011*

Values presented as mean ± standard deviation (SD)/*significant values.

**Table 5:** Food intake values before (time 1) and after (time 2) treatment with water (G1 and G2) and acerola juice (G3 and G4).

	Groups				*P* value
	G1	G2	G3	G4	
Food intake (time 1)	120.0 ± 58.2	139.5 ± 15.5	115.5 ± 11.1	127.0 ± 39.1	.0732
Food intake (time 2)	112.8 ± 38.8	133.2 ± 11.6	118.7 ± 5.9	123.8 ± 27.2	.0670
*P* value	.0661	.1927	.0603	.1003	

Body weigh (time 1)	248.6 ± 49.9	255.5 ± 59.7	268.6 ± 61.3	282.12 ± 56.6	.0501
Body weigh (time 2)	305.5 ± 89.2	326.2 ± 48.2	329.6 ± 61.9	338.26 ± 76.2	.0578
*P* value	.0781	.0689	.0789	.0798	

Values presented as mean ± standard deviation (SD).

**Table 6:** Food intake values on the first day of pregnancy (time 1) and on the day preceding the birth of their offspring (time 2) in the non diabetic (NDM) and in diabetic dams (DM).

	Groups		*P* value
	Dam NDM	Dam DM	
Food intake (time1)	120.0 ± 37.1	139.5 ± 25.3	.0732
Food intake (time 2)	142.8 ± 39.4	153.2 ± 59.5	.0670
*P* value	.0661	.1927	

Body weigh (time 1)	306.6 ± 59.4	280.5 ± 39.7	.0652
Body weigh (time 2)	429.5 ± 45.7	430.9 ± 54.2	.0668
*P* value	.0381*	.0299*	

Values presented as mean ± standard deviation (SD)/*significant values.
